# Cognitive, functional and affective effects of a multi-task training in nursing home residents: results from a randomized pilot study

**DOI:** 10.1007/s40520-026-03330-6

**Published:** 2026-01-30

**Authors:** Clara Burgo-Beiro, David Facal, Carlos Dosil-Díaz, Alba Felpete, Jhilenia Villamide-Gesto, María Campos-Magdaleno

**Affiliations:** 1 Universidade de Santiago de Compostela, Santiago de Compostela, 15705 A Coruña Spain; 2 Department of Developmental Psychology, Universidade de Santiago de Compostela, Santiago de Compostela, 15705 A Coruña Spain; 3 Institute of Psychology (IPSIUS), Universidade de Santiago de Compostela, Santiago de Compostela, 15705 A Coruña Spain

**Keywords:** Dual-tasking training, Cognitive training, Ageing intervention, Long-term care center, Self-esteem

## Abstract

**Background:**

Dual-task (DT)-based training programs are non-pharmacological interventions that potentially improve cognitive, physical-functional and affective processes in older adults. In the specific context of nursing homes, typically characterized by impoverished environmental stimuli, sedentarism and affective alterations, these interventions remain poorly studied.

**Aims:**

The aims of this randomized pilot study were to evaluate the feasibility of implementing the Resi-TaD DT training program in long-term care centers (LTCCs) and to evaluate the impact of the program on cognitive, physical-functional and affective processes in elderly residents.

**Methods:**

An intervention program was designed within the framework of the multitasking paradigm; the physical and cognitive components were used to monitor the level of difficulty of each combination of tasks, which included triple-tasks. Study participants (*n* = 94) were assigned to an Intervention Group (IG) or a waiting-list Control Group (CG), and cognitive (MoCA, DT performance), physical-functional (TUG and handgrip strength) and affective (depressive symptomatology and self-esteem) measures were evaluated before and after the intervention. Dual-task (DT)-based training programs are non-pharmacological interventions that potentially improve cognitive, physical-functional and affective processes in older adults. A General Lineal Model with Repeated Measures was used to assess differences and interactions between IG and CG performance at each time. Participation and dropout rates were also considered.

**Results:**

The multi-task intervention was feasible and well accepted by LTCCs users. In the IG, improvements in general cognition and DT performance but no significant differences in TUG or handgrip strength were observed. A subtle but non-significant improvement in affective measures was observed.

**Conclusions:**

A 10-session intervention involving heterogeneous activities can enhance cognitive function and potentially promote transfer effects to other DTs. However, the intervention is not sufficient to achieve significant physical-functional or affective benefits.

## Introduction

The number of older adults who reside in long-term care centers (LTCCs) is increasing worldwide due to longer life expectancy and need for care [1]. In nursing homes, institutionalized older users spend an average of 11.6 h a day on non-stimulating sedentary activities, such as watching TV and napping [2], and they spend long periods in bed [3, 4]. In addition, the high level of care provided in nursing homes reduces the opportunities for users to participate in activities of daily living (ADL) (e.g., doing laundry, preparing meals, transportation, or personal care), which may further reduce the level of independence of residents [2]. The lifestyle in LTCCs is thus more impoverished than in other care models or community life, with fewer opportunities to engage in stimulating activities that promote cognitive reserve [5]. In Galicia (north-western Spain), nursing homes residents are predominantly of advanced age, female, highly dependent and with a high incidence of cognitive impairment, in some cases without dementia being diagnosed [6].

Some interventions that aim to reduce sedentary behaviors in LTCCs by providing opportunities to engage in physically and cognitively stimulating activities are based on multitasking [7]. The multi-tasking paradigm combines motor and/or cognitive tasks simultaneously, rather than separately, most commonly in two activities (dual tasking, DT). DT is common in daily living activities (e.g., walking while talking, eating while listening to the news) but the ability to distribute the cognitive resources required to attend to simultaneous demanding tasks tends to decline with age [8–12] owing to a reduction in executive attention, resulting in poorer performance of dual tasks [13].

The findings of multi-tasking interventions have shown that DT performance can be enhanced by DT intervention programs [14–16], with documented improvements in cognitive function [15, 17, 18] and gait parameters [14, 15, 17] in community-dwelling healthy adults, in people with Parkinson’s disease (PD) without dementia [19] and in people with Alzheimer’s disease (AD) dementia [20, 21]. Two meta-analyses focusing on cognitively impaired older adults revealed that DT training interventions enhance cognition, gait/functional mobility and depressive symptomatology [22, 23].

Information on the effects of DT interventions in nursing homes remains scarce. Rezola-Pardo et al. [24] found that the effect of a combination of multicomponent training and cognitive load was no better than single multicomponent training, although the program design focused on physical exercise and the cognitive load of DT training was added to the physical training a posteriori. A similar approach was used by Rondao et al. [25], who incorporated computerized cognitive training into the aerobic and strength exercises in nursing home residents with mild cognitive impairment. This study found that the physical and functional performance was better in the experimental group than in the control group. Following a similar approach, in the PRO-CARE research project, Bischoff et al. [26] and Cordes et al. [27] developed interventions that include DT among other single physical workout and cognitive tasks, in LTCC participants able to walk and chair-bound participants, respectively. Both studies found improvements in motor functions, cognitive performance and depressive symptomatology of participants relative to waiting-list controls. Using a different approach, Yoon et al. [28] studied the effects of a DT training program based on traditional Japanese games. The SYNAP program tries to stimulate brain activity using both motor tasks (i.e., rock-paper-scissors, colored ball games) and cognitive tasks (i.e., simple calculations, memory word games). The authors observed significant improvements in cognitive status and physical function in the experimental group of LTTCs residents in comparison with an equivalent no-intervention group.

Performance in DT is influenced by a wide range of cognitive, physical-functional and psychosocial variables in old age [7, 29], including factors related to mental health and emotional well-being. Affective alterations are common in institutional settings, where more than half of the residents have some degree of depressive symptomatology [30]. Symptoms of depression in LTCCs users have been negatively related to preservation of functional capacities, ADL and autonomy [30, 31]. On the other hand, LTCC residents have been shown to have lower levels of self-esteem than elderly people living in their own homes [32], even those who live alone [33]. Levels of self-esteem have also been positively correlated with independence in ADL in nursing homes [34]. However, fewer than one-third of the interventions conducted in nursing homes assess the impact on mental health, and only about a quarter report positive outcomes [35]. Specifically regarding DT training programs, Ye et al. [22] reported that, of the 20 intervention programs included in a meta-analysis on older adults with cognitive impairment, only five examined the effects on depression, with results suggesting a reduction in depressive symptoms; however, none of the studies provided information about the application settings (institution, community, or home-based). We only found three studies analyzing the impact of DT interventions on the mental health in LTCCs users, with mixed results. While Bischoff et al. [26] and Cordes et al. [27] found significant improvements in depression, Rezola-Pardo et al. [24] reported a non-significant tendency for anxiety and depression to increase after DT training. We did not find any studies evaluating the impact of a DT training programs on self-esteem among older adults in LTCCs or in the community.

Multi-tasking training is a non-pharmacological intervention designed to stimulate and enhance physical, functional and cognitive parameters, as well as potentially emotional parameters, in older adults. This approach is particularly important in LTCCs, in which residents typically have sedentary lifestyles, functional and cognitive impairment, and/or some degree of frailty that limits their independence [36]. Most intervention programs based on multitasking focus on one physical task pus one cognitive task under DT conditions. However, other possibilities exist, such as the simultaneous performance of two cognitive tasks (cognitive + cognitive), two physical tasks (physical + physical) and even triple-tasking (TT) (i.e., performing three tasks at the same time). TT represents a particularly demanding combination that compels individuals to operate at the limits of their resources. This can be clinically meaningful, given that some daily activities require performance of more than two tasks simultaneously. This highly demanding multitasking format is sensitive to cognitive impairment and frailty in older adults [7, 36]. In multi-tasking training interventions, it can also be important to monitor the level of difficulty of each task combination, to ensure they are challenging enough without the training sessions causing frustration.

Feasibility studies help to determine whether particular programs can be implemented in large scale trials, taking into account the complexity of the care context, the needs of older residents and the complexity of the program design. Beyond acceptability and resource requirements, this type of study must consider the importance of sustained engagement and adherence throughout the intervention. In this regard, Sakurai et al. [37] found that a multidomain intervention was particularly effective for older adults with over 70% attendance at exercise sessions compared to a nonadherent intervention group.

We conducted this study to explore the feasibility and potential use of a multi-tasking training program among LTCC users, regarding the following: (1) acceptance of the intervention content and assessment protocol; (2) adherence to and motivation for training sessions; and (3) any difficulties experienced by the nursing home staff when implementing the program. We also analyzed the impact of the intervention on DT performance (as the primary outcome) and on physical performance, cognitive functioning and affective status (as secondary outcomes), in a sample of very old residents of nursing homes. The program includes a variety of tasking combinations (physical + cognitive, cognitive + cognitive, physical + physical, and TT) and monitors the intrinsic complexity of each combination through a progressively challenging design.

## Materials and methods

### Study design

This trial was designed and reported in accordance with the CONSORT 2010 statement [38]. A randomized multicenter pilot trial was conducted over three-month periods in ten nursing homes (nine medium-sized and one large home) in Lugo (Galicia, NW Spain), between October 2023 and May 2024. While previous studies in LTCCs have included dual physical-cognitive tasks, our pilot design considered the physical and cognitive components of the DTs separately, but complementarily, and also incorporated TTs.

Data collection was carried out by trained psychogerontologists. Staff at the centers (occupational therapists, psychologists) were responsible for randomly assigning participants to the groups (by pseudonymized form and using www.random.com) and conducting the intervention sessions. The intervention group received DT training sessions once a week for 10 weeks, in 50-minute sessions with groups of approximately 6 participants. The control group was placed on a waiting list for the intervention and continued performing the usual activities in the LTCCs during the trial. We used a relatively brief intervention to enhance the study feasibility, particularly with respect to operational constraints in nursing homes, as well as the participants’ acceptance of adherence to the intervention.

All participants were assessed before and after the intervention at approximately the same time of day, to avoid any variations due to circadian rhythm, in a quiet, well-lit room in each nursing home. To guarantee a double-blind process, the evaluator only received the participants’ codes when carrying out pre and post intervention assessments and was not aware whether the participants belonged to the control or intervention group. The statistical analyses were carried out by a researcher who was blinded to the meaning of the group variable.

The study received the approval of the Bioethics Committee of the University of Santiago de Compostela (reference number USC70/2022). It was conducted in accordance with the Declaration of Helsinki, revised in Brazil 2013. Participants did not receive any economic compensation. Written informed consent was obtained from all individuals prior to their participation in the study, and the staff at the LTCCs informed the families and/or legal guardians about the aims of the research when needed.

## Participants and recruitment

**Fig. 1:**
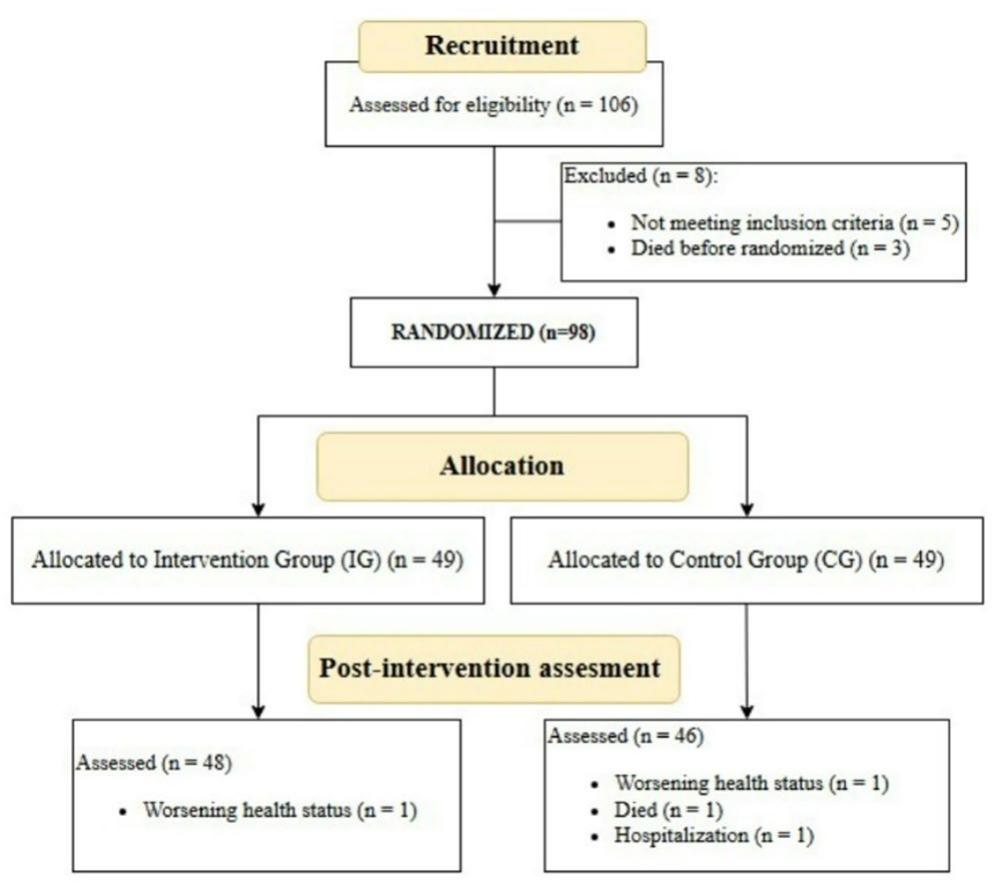
Participant flow-chart

Among 106 participants initially proposed by the LTCC staff, 98 were recruited after checking eligibility criteria in the pre-intervention assessment. The participants were randomly assigned to the Intervention Group (IG) or Control Group (CG) in a ratio of 1:1. Finally, a total of 94 long term institutionalized older adults completed the post-intervention assessments (IG = 48; CG = 46) and were included in the final sample and statistical analysis (see Fig. 1).

The inclusion criteria were as follows: age 60 or older; cognitively healthy or mild to moderate cognitive impairment that allowed the participation in the program according to the records of cognitive status assessments carried out by LTCCs using the Mini-Mental State Examination; ability to stand up from a chair independently; preserved walking ability (able to walk independently or with an aid for 10 m). The exclusion criteria were diagnosis of dementia and cognitive disability or sensory impairment that would prevent correct implementation of the intervention or assessments.

## The Resi-TaD: dual task-based training program for nursing homes

Resi-TaD (Dual-Task Intervention Program in Nursing Homes, for its Spanish acronym) is an innovative program that offers advantages over previous DT interventions for older adults. In most previous research, training programs have been based on combining a physical and a cognitive task (physical + cognitive DT activity). Resi-TaD also uses less common task combinations, such as two physical tasks (physical + physical) or two cognitive tasks (cognitive + cognitive). For example, tandem standing combined with receiving and throwing a ball or finding the exit in a maze puzzle combined with a word association game. Moreover, the selected tasks are varied, avoiding repetitive performance of the same task in the same or consecutive sessions. Second, Resi-TaD includes TT in later sessions as part of a protocol including increasing levels of difficulty.

Resi-TaD is not based on a physical training program with added cognitive tasks, a common approach used in the literature. This program was specifically designed within the framework of the multitasking paradigm, following the taxonomy described in McIsaac et al. [39]. For each single task, we considered the two axes that define the task difficulty by monitoring the level of challenge: the task complexity, or the number of components and the attentional demands; and the task novelty, or the individual’s own previous experience of the task [39]. For example, the reverse repetition of a digit sequence is generally a rare task (high novelty) that also involves several intrinsic components and requires a high attentional demand (high complexity), and it is therefore considered a difficult task. By contrast, answering simple yes-or-no questions (e.g., “is breakfast consumed at night?“, “are umbrellas used for the rain?“) is a very frequent task (lower novelty) that contains few intrinsic elements (low complexity), and it is therefore considered an easy task. Alternatively, standing is a very frequent task (low novelty) with few intrinsic components (low complexity), and it is considered an easy task. However, tandem walking (placing one foot directly in front of the other) is a rare task (high novelty) with more intrinsic components (high complexity), and it is considered difficult. By combining these four ST in a DT condition, we can construct a very easy DT (standing + answering simple questions), a very difficult DT (tandem standing + reverse digit sequence), and two DT of intermediate difficulty (tandem walking + simple questions, standing + reverse digit sequence).

Taking this taxonomy into account enabled us to design a program of increasing difficulty, both over the course of the weeks and within each individual session. Thus, TTs were included during the final sessions, as these are the most demanding. When designing TTs, it is essential to consider the compatibility of the tasks (for example, counting backwards aloud is incompatible with semantic fluency, and walking is incompatible with tandem standing) and also to carefully assess the difficulty of the combinations to ensure that execution is feasible, given the substantial attentional resources required. For example, in a circle of 6 participants in tandem stance, passing the ball to another random person while saying his/her name is a TT with compatible actions, and the difficulty of each task is balanced. This is the first evidence, as far as we know, of the use of TTs in a training program for older people.

Each of the 10 sessions is designed to be delivered in approximately 50 min, following a common structure. The sessions begin with a 10-minute welcome period, during which the characteristics of the session are explained, and large muscle groups are mobilized. This is followed by the central part of the training which lasts approximately 30 min. The combination of tasks is always presented in the same order: physical + cognitive, physical + physical, cognitive + cognitive. The final ten minutes of each session are used for cooling down, including a group discussion to share impressions of the experience and an invitation to return the following week. The program sessions are summarized in Table 1.

**Table 1:** Resi-TaD summary of sessions

	Physical + cognitive tasks	Physical + physical tasks	Cognitive + cognitive task
Session 1	Walking + narrating an important event. Walking + phonological fluency.	Standing + eyes closed. Tandem standing + eyes closed.	Phonological fluency + maze puzzle. Cutting out shapes + telling a story.
Session 2	Walking + narration of an important event. Walking + naming the opposite (non-weight-bearing) foot.	Standing + eyes closed. Standing + throwing (or catching) a ball.	Cutting out shapes + telling a joke. Coloring + yes-or-no questions.
Session 3	Tandem walking + counting backwards. Walk backwards + counting in twos.	Standing + throwing (or catching) a ball. Walking + holding a glass with water.	Origami + counting by twos. Cancellation task + repeating digit sequences.
Session 4	Walking + naming the opposite (non-weight-bearing) foot. Walking backward + counting backwards.	Walking + holding a glass with water. Walking + carrying a tray with a glass with water.	Coloring + semantic fluency Cutting out shapes + describing a recipe.
Session 5	Walking + repeating backward digits. Tandem walking + reverse digit sequence.	Walking + imitating movements. Walking + raising opposite arms.	Cancellation task + riddles. Cancellation task + sing a song.
Session 6	Walking + estimating times. Sitting and standing + low-difficulty calculations.	Tandem standing + throwing (or catching) a ball. Walking + imitating movements.	Cutting out shapes + phonological fluency. Cutting out shapes + word game.
Session 7	Walking backwards + explain the use of different objects. Sitting and standing + medium-difficulty calculations.	Walking + throwing (or catching) a ball Throwing a ball with one hand + catching a ball with other hand	Coloring + riddles. Spot the difference + recalling a list of words.
Session 8	Throwing a ball + semantic fluency. Throwing a ball + name who catch the ball.	Walking forward + throwing a ball. Tandem walking backward + switching a ball between hands	Maze puzzle + answering questions. Origami + counting in fives.
Session 9	Throwing (or catching) a ball + asking (or answering) a question. Tandem standing + throwing a ball + naming person who catches the ball.	Tandem standing + holding a glass with water Tandem walking + carrying a tray with a glass with water.	Coloring + completing proverbs. Repeating sentences + coloring + tapping rhythms with feet.
Session 10	Most engaging tasks from the previous sessions		

## Measures

To assess the feasibility of the Resi-TaD in terms of acceptance, the participation and dropout rates were recorded. The feasibility of the assessment protocol was determined by completion of the measurement sessions (pre and post intervention). To evaluate adherence to the program and to collect information on the difficulties that arose during its implementation, reports were requested from the staff at all centers.

To assess the impact of the training program, all participants underwent cognitive, physical and affective assessment before and after the intervention. Apart from these key domains, demographic characteristics (age, sex and schooling) were collected at baseline.

General cognition was assessed using the Montreal Cognitive Assessment (MoCA) [40–42], selected for its capacity to monitor subtle cognitive changes. Performance under DT conditions was also considered using two cognitive tests not included in the intervention program: a tracking task, and an ideational fluency task. The tracking task, described by Della Sala et al. [43], requires participants to use a pencil to follow a path composed of circles connected by lines on a sheet of paper. The final score is the number of circles reached in one minute. For the ideational fluency task, two prompts were used based on actions or behaviors that people can perform [44]: participants were asked to say what defines a good citizen and what defines a good mayor in one minute. Both tasks were performed in the single condition (single-task, ST) and combined in DT (counterbalancing the components). To calculate the difference in task performance between the ST and the DT conditions (DT costs), we used the formula proposed by Montero-Odasso et al. [45]: [(DT performance – ST performance)/ST performance].

Two measures were selected to evaluate physical-functional performance. First, the Timed Up and Go (TUG) test [46], which consists of getting up from a chair, walking 3 m, turning around, and sitting down again. This test provides a measure of overall mobility, consisting of the time it takes the participant to complete a task. The second measure was a handgrip strength measure (related to both weakness and mobility outcomes [47]), assessed using a handheld dynamometer.

Finally, the affective measures used were depression and self-esteem tests, related to quality of life and well-being. To assess depressive symptoms, the abbreviated 15-item Geriatric Depression Scale [48, 49] was used. In this test, higher scores indicate greater depressive symptomatology. To assess the participants’ global self-esteem, the Rosemberg Self-Esteem Scale [50, 51] was administered by an interviewer. In this case, higher scores indicate greater self-esteem and vice versa, so that the interpretation is the opposite of the GDS-15 scores.

## Statistical analyses

Participation and dropout rates were considered in order to measure the trial feasibility. Group differences in demographics were analyzed using a chi-squared test for sex (categorical measure) and a Student’ t test for age and years of schooling (continuous measures). General Linear Model (GLM) were used to compare group differences in primary (cognitive) and secondary (physical-functional, affective) outcomes, and GLM repeated measures (GLM-RM) were used to compare performance between groups at each time. Primary and secondary measures were the dependent variables included in the within-subject, with two levels (pre- and post-assessment). The factors considered in the analysis were performance at each time point (pre- and post-intervention) and group (IG and CG).

Considering the wide range of variability in baseline cognitive status and chronological age, sensitivity analyses were conducted in order to confirm the effects of the intervention beyond the influence of these variables. Specifically, the GLM-RMs were replicated by including the MoCA score in the pre assessment as covariate to control for the effect of cognitive status at baseline. The GLM-RMs were also replicated by including the factors cognitive status (cognitive status above 15 in the MoCA test, or equal to or below 15 in the MoCA test) and age (age below 86 years, or equal to or above 86 years).

The statistical tests were conducted using IBM SPSS Statistics, version 29.0.2.0.

## Results

Of the 98 participants initially included in the study (from both the IG and the CG), all completed the program and the assessment sessions, except for four individuals (2 because of worsening health status, 1 because of hospitalization and 1 who died) (see Fig. 1). Lack of motivation was not recorded in any of the dropout cases. Regarding adherence to the content of the Resi-TaD sessions in the IG, the staff reported high attendance rates, with very occasional absences (e.g., because of medical appointments). The staff noted the attractiveness of the program: participants attended punctually, had a positive attitude and enjoyed the activities. Finally, staff reported no difficulties, incidents or barriers regarding implementation of the sessions. No adverse events, falls or harm occurred during the training sessions or the assessments. All participants were equally able to perform the exercises according to the intervention staff.

The main demographic characteristics are presented in Table 2; there were no significant differences in age, years of schooling or sex distribution between groups.

**Table 2:** Demographics with mean values (and standard deviations) for groups and total sample, with mean values (Student’s t) and frequencies (chi-squared) comparisons between groups

	Intervention Group (*N* = 46)	Control Group (*N* = 48)	Total sample (*N* = 94)	χ2/t
Age	83.02 (8.3) Range: 64–94	83.83 (8.04) Range: 66–100	83.44 (8.10) Range: 64–100	− 0.48
Sex	52.17% women 47.83% men	56.25% women 43.75% men	54.26% women 45.75% men	0.16
Years of schooling	7.37 (4.01)	7.65 (3.28)	7.51 (3.64)	− 0.37

**Fig. 2:**
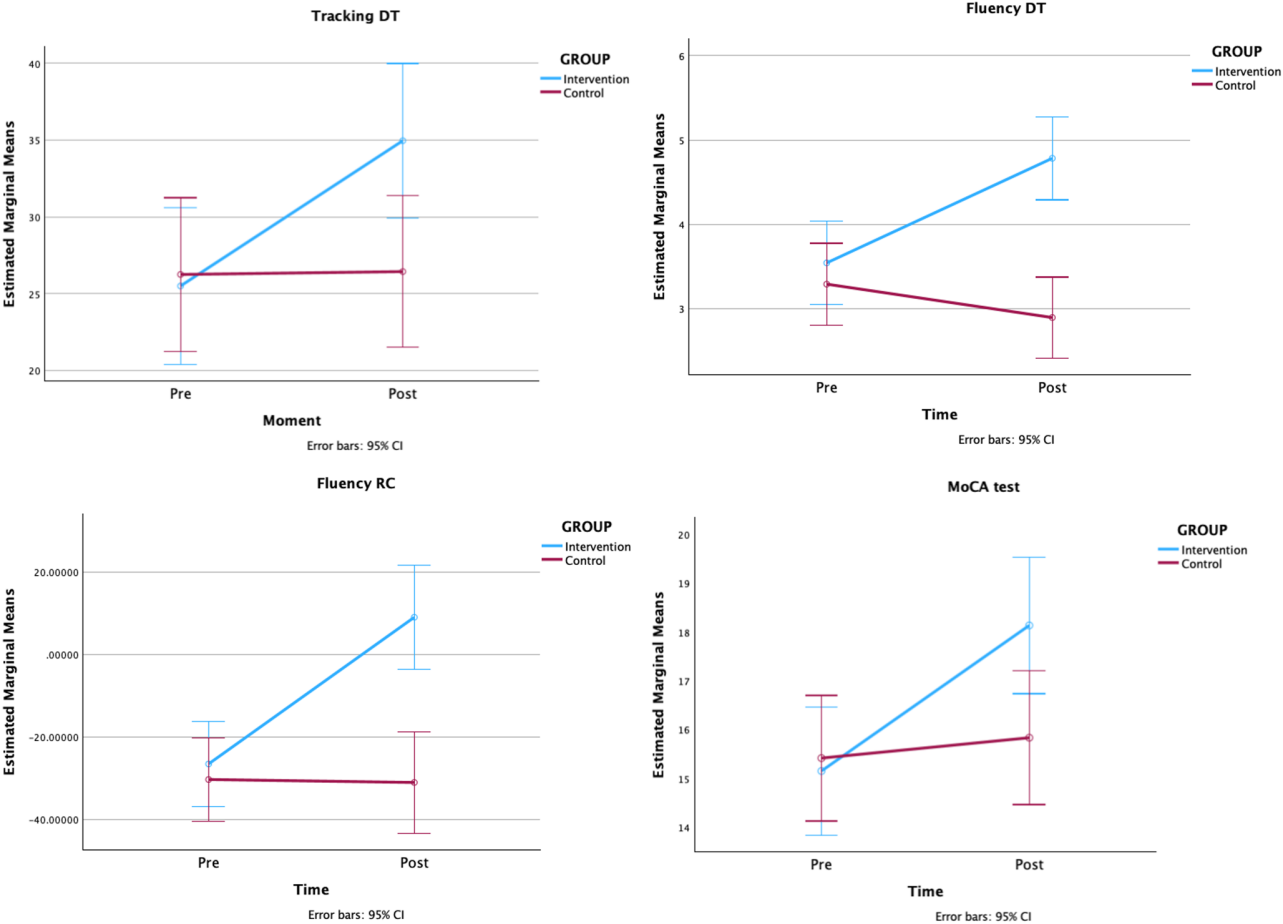
Estimated marginal means of GLR-RMs with significant interaction between time and group factors

Descriptive statistics for the primary and secondary measures and GLR-RM results are shown in Table 3. Regarding the DT tasks assessed, the interaction between the within-subjects pre and post intervention assessment and the group factor showed significant improvements in the DT conditions (tracking and fluency), with the intervention group showing significant improvement in the second assessment, relative to the control group, and in the response costs in the fluency task. There was also a significant time-group interaction in the MoCA tests results, with the IG improving in the post-intervention assessment. The effect of GLM-RMs with significant time x group interactions is shown in Fig. 2. The TUG measure decreased in the intervention group after participation in the Resi-TaD, whereas the CG showed similar results, but the interaction was not significant. These changes were accompanied by a non-significant improvement in the IG in the GDS scores, compared with a slight impairment in the CG, and with non-significant increase in the RSES scores in the IG, compared with absence of changes in the CG.

**Table 3:** Mean values (and standard deviations) in the pre and post assessment for intervention and control groups, and time x group interactions

	Intervention Group (*N* = 46)		Control Group (*N* = 48)		F (1,92)	*p*	η^2^_*p*_
	Pre	Post	Pre	Post			
Tracking ST (total circles)	37.89 (20.26)	44.35 (20.34)	39.63 (23.25)	39.81 (23.25)	3.13	0.08	0.03
Tracking DT (total circles)	25.5 (16.06)	34.96 (18.73)	26.25 (18.64)	26.44 (15.6)	9.33	**< 0.01**	**0.09**
Tracking RC	−32.95 (19.77)	−18.72 (23.81)	−34.74 (22.81)	−31.85 (23.31)	3.63	0.06	0.04
Fluency ST (total words)	4.93 (1.53)	4.43 (1.47)	5.00 (1.47)	4.44 (1.76)	0.03	0.87	0.00
Fluency DT (total words)	3.54 (1.85)	4.78 (1.89)	3.29 (1.53)	2.9 (1.45)	21.41	**< 0.001**	**0.19**
Fluency RC	−26.55 (39.09)	9.01 (40.53)	−30.33 (31.19)	−31.05 (45.20)	10.53	**< 0.01**	**0.10**
MoCA score	15.15 (5.10)	18.13 (4.84)	15.42 (3.81)	15.83 (4.71)	13.73	**< 0.001**	**0.13**
TUG (secs)	22.11 (10.7)	19.87 (11.23)	25.05 (14.65)	24.90 (14.51)	1.47	0.23	0.02
Handgrip strength (kg)	19.56 (7.76)	20.29 (8.18)	18.97 (6.4)	18.65 (6.79)	3.09	0.08	0.03
GDS score	3.91 (2.67)	3.52 (6.05)	3.98 (2.97)	4.4 (3.41)	0.78	0.38	0.01
RSES score	31.80 (6.09)	33.17 (6.90)	32.21 (5.15)	32.67 (5.46)	0.49	0.49	0.01

ST = Simple Task; DT = Dual Task; RC = Response Cost; MoCA = Montreal Cognitive Assessment; TUG = Timed Up and Go; GDS = Geriatric Depression Scale; RSES = Rosemberg Self-Esteem Scale

**Fig. 3:**
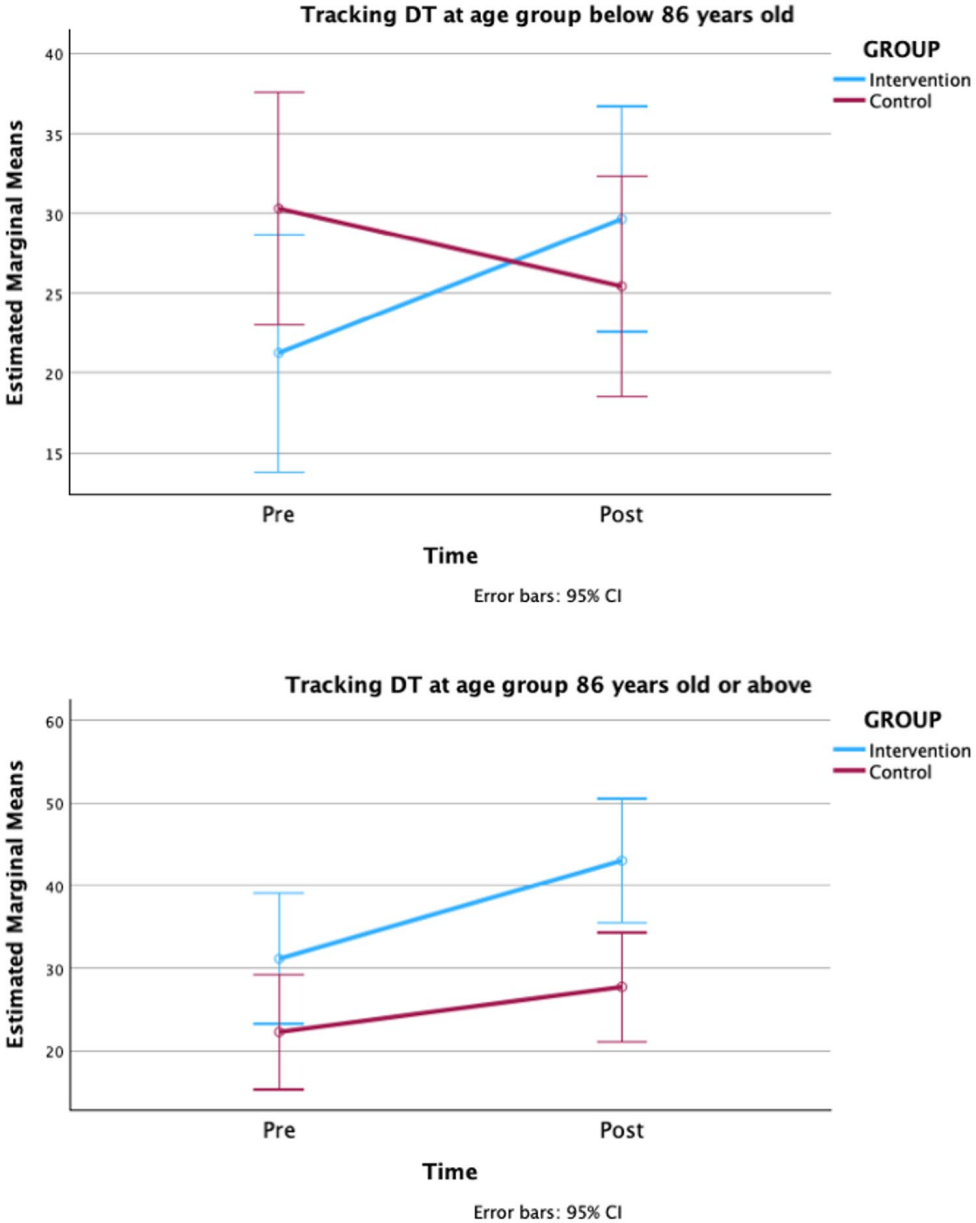
Estimated marginal means of the time x group interaction in both age groups, in the GLR-RM with the tracking DT performance as the dependent variable

When the baseline MoCA score was included as a covariate in the analyses, no significant effect of the covariate was found in any of the GLR-RMs, and the significant differences reported above remained unchanged. Inclusion of cognitive status and age as factors in the GLM-RMs led to a significant interaction between time and age group in the GLR-RM with tracking DT performance as the dependent variable (F = 4.72; *p =* 0.03; η^2^_p_ = 0.05). The time x group interactions in both age groups for this dependent variable were plotted in Fig. 3, showing that an interaction between time and the intervention group occurs in the younger age group.

## Discussion

Resi-TaD is an intervention training program designed within the framework of the multi-tasking paradigm, including a wide variety of tasks and combinations. The present study was designed to evaluate the feasibility of the Resi-TaD program, the data collection protocol and the potential effects of the program on cognitive functioning, physical performance and affective status in a sample of very old residents in LTCCs. The high participation rate and level of motivation among individuals in the intervention group indicate that Resi-TaD is attractive, enjoyable and well-tolerated among nursing home residents. No difficulties, incidents or harm were reported, demonstrating that the program can be safely implemented by the staff. Therefore, no changes are anticipated in the session content or in the evaluation system for the future implementation of the program.

The GLR-RM results showed significant improvements in global cognition (measured with MoCA test) and in DT performance in the IG after the training, relative to the same measures in the CG, which remained stable over the study period. Improvements in DT performance were observed in both simultaneous tasks. IG participants increased the number of correct responses generated in the ideational fluency task and the number of circles reached in the tracking path after training. The results also showed a significant reduction in ideational fluency DT costs when the task was performed simultaneously with tracking. Our finding are consistent with those of previous studies in different samples of older adults, where enhancement of global cognition has been identified by meta-analysis [22, 23, 52], with small to medium size effects reported, and well-documented positive effects on DT performance due to reductions in response costs [14–16, 19, 21, 52]. In the context of nursing homes, preliminary findings follow the same trend. DT interventions tend to maintain or improve general cognitive function [24, 25, 27, 28]. Only Rezola-Pardo et al. [53] analyzed the impact of the multi-tasking intervention in nursing homes on DT performance, reporting a reduction in DT costs. The present study is the first to report significant cognitive improvements both in general cognition in DT performance in a DT-based IG compared with a waiting-list CG, thus providing preliminary evidence of the effectiveness of this type of program even in very old adults living in nursing homes.

In our study the combinations of tasks are different from those used in the assessment protocol. IG participants showed significant improvements in DT performance (lower DT costs in ideational fluency and better performance on each task in simultaneous execution) after training. This suggests a certain degree of transfer, allowing the gains to be generalized to a different task combination. Although the transferability of DT-based training compared to other types of interventions in ageing is still being investigated, it has been observed that interventions involving heterogeneous activities can promote earlier transfer [54]. Our relatively short intervention, based on a variety of activities, indicate that DT training involving a wide range of tasks may be a key factor for generalizing results to other activities, particularly regarding cognitive and DT performance. In summary, our findings suggest that general cognitive function and multitasking capacity can be trained and enhanced through short intervention programs, and improvements can be transferred, particularly within nursing home settings. Given that the available evidence shows that the effect of DT training is associated with a reduction in activation of the regions involved in this processing after training, which is interpreted as an indicator of neural efficiency [54–56], the improvement in participants’ TD performance may be related to greater efficiency of task execution, which would prevent over-recruitment, while an improvement in overall cognitive status may be associated with more efficient use of processing resources.

Regarding the physical-functional parameters assessed, by the TUG and the handgrip strength measures, our results did not show any significant improvements after training. Previous intervention studies have reported benefits in various gait-related parameters (gait speed, stride length, etc.) and other physical-functional measures (handgrip strength, endurance, cardiorespiratory capacity, etc.) in the general old population. However, these studies have one or more of the following characteristics: (1) they are grounded in a multicomponent physical training program of a certain intensity (e.g. aerobic exercises, strength, resistance training, speed training,.), to which a cognitive load is added to convert it into DT training [14, 15, 20, 25, 53, 58]; (2) they rely on the repeated use of walking as the physical task during the intervention, combining it with simultaneous cognitive tasks and subsequently measuring their impact on gait parameters [14]; (3) they include dedicated time for performing physical training activities of a certain intensity in addition to DT training [26, 27, 59]. Within this framework, in which DT programs place a strong emphasis on fitness, physical-functional benefits are more likely to be achieved. By contrast, the physical tasks included in the Resi-TaD program are not fitness-based and therefore do not focus on musculoskeletal training. Rather, they are based on low-intensity tasks related to movement, balance and coordination.

In the specific context of nursing homes, findings of studies that meet the characteristics described in points 1 and 3 above have shown mixed outcomes regarding handgrip strength. Bischoff et al. [26] and Cordes et al. [27] reported improvements after DT intervention, whereas Rezola-Pardo et al. [53] did not observe any significant changes. Conversely, better results were not obtained in the up-and-go capacity in the DT intervention group relative to participants who only received multicomponent single physical training [24, 53] or with a control group [25]. Meanwhile, Yoon et al. [28] used a DT training program similar to Resi-TaD in which tasks may involve movement and/or coordination but are not associated with fitness training. In this case, preliminary results from a small sample did not show any improvements in handgrip strength, but a significant reduction in the time taken to complete the TUG test. Given the absence of any improvements in the physical measures, we hypothesized that the effects of DT-based intervention programs on handgrip strength may depend more strongly on the intensity of the physical training than on the ability to coordinate attentional resources across components on the multitasking conditions. Moreover, our TUG test findings are consistent with findings of previous studies conducted with LTCCs participants and involving high-intensity physical activities; however, they contrast with those obtained in the study by Yoon et al. [28], which included low-intensity physical activities. Further research should be carried out to examine the effects of DT interventions on physical functioning, particularly with the ability to perform up-and-go tasks, in nursing home settings which are typically characterized by sedentary behavior and limited opportunities to engage in ADL, regardless of the physical intensity of the intervention program.

Finally, our results did not show any significant changes in depressive symptomatology or self-esteem. As noted in the introduction, mental health is associated with multi-tasking performance capacity; however, this area remains underexplored. Although there is evidence of a reduction in depressive symptoms after DT interventions in the general population of older adults [18, 22], findings in nursing home settings are scarce and mixed. Cordes et al. [27] reported a reduction in depressive symptoms, whereas Rezola-Pardo et al. [24] did not observe any significant changes, although they noted a non-significant trend towards worsening of depressive and anxiety symptoms. While our findings did not reveal any significant differences in affective measures, a slight increase in self-esteem and a small reduction in depressive symptomatology were observed in the IG (although neither of these differences were significant). Although several factors have been identified as potentially being related to the effects (or lack thereof) of DT training programs on affective measures (e.g., frustration due to task demands, opportunities for social interaction) [22, 24], our findings suggest that the use of well-tolerated tasks with gradually increasing difficulty in group-based sessions may not be sufficient to achieve significant affective benefits.

The current results show that the intervention promoted transfer to other DTs, since the DTs included in the evaluation differed from those included in the intervention. Regarding transfer of the findings to daily life, Cordes et al. [27] analyzed the impact of an intervention including DT on a sample of institutionalised participants with walking difficulties. This intervention involved specific training in basic activities involving everyday objects, such as towels, newspapers and cups, in both single- and dual-condition settings. The results showed that the Barthel index score increased in the intervention groups, whereas the score decreased in the control group. In contrast to the previous study, Bischoff et al. [26] did not conduct specific training in basic activities and observed a decrease in Barthel index scores in both the intervention and control groups. Building on the study by Yokoi et al. [18] -which allowed participants to individually decide the type of task combinations performed during training, always including ADL - it is possible that affective benefits are also related to the individualization of tasks and the provision of more ecologically alternatives of combination. Thus, future research must examine the effectiveness of DT intervention based on ADL in nursing homes, especially with regard to affective measures and to their impact on the daily lives of residents.

As mentioned above, we selected a relatively brief intervention to enhance the study feasibility, in light of the limitations in the context of care homes. Regarding the duration/frequency of intervention, in a meta-analysis in older participants living in the community, Wollesen et al. [52] found that to improve global cognitive functioning the total training duration should range between 8 and 104 h, with the frequency differing between one or two times per week with 30, 60–90 min sessions. These authors recommend at least 60 min training per week and a total duration of 12 h in order to gain positive effects on the different cognitive domains. In a meta-analysis in participants with cognitive impairment, Ye et al. [22] suggested a frequency of 1–2 sessions weekly, each lasting more than 45 min but no longer than 60 min, for a period of 13–26 weeks according to the results of a subgroup analysis. Similarly, Yu et al. [23] showed that DT training of 30–60 min/session two-three sessions/week, with total training lasting 18–30 h, had a positive effect on cognitive and functional measures in people with cognitive impairment. Accordingly, our intervention would need to be implemented more frequently in order to achieve functional effects.

Given that long-term care facilities often constitute socially impoverished environments, the social interaction fostered by the program may enhance intervention outcomes by increasing motivation and strengthening group dynamics. Future research should therefore assess the impact of the program on residents’ social interactions. Other changes for larger RCTs in the future may include the development of tasks that are more closely related to daily life (e.g. buttoning clothes, pouring liquids, cutting fruit and vegetables), and as personalised as possible. The possibility of increasing the number of sessions and, if feasible, their frequency, will also be explored.

## Conclusions

We conclude that the Resi-TaD is an easily implemented, participant-friendly program, that does not cause frustration in participants. The advantages of this program include (1) it is designed to be implemented by the staff at centers without the need for external support; (2) it uses varied tasks, including uncommon combinations (cognitive + cognitive and physical + physical); and (3) it assesses the intrinsic difficulty of each task in order to monitor the progression difficulty, which includes a TT in later sessions. However, an important limitation is that the task used are “laboratory-based” and do not include actions commonly performed in daily life. In summary, Resi-TaD is a feasible intervention program for nursing home settings that provides opportunities to engage in stimulating activities. The results demonstrate that a relatively brief intervention (one 60-minute session per week over 10 weeks) involving heterogeneous activities is sufficient to lead to cognitive improvements, although no significant effects were observed at the physical-functional or affective levels. Future research into multi-tasking training in LTCCs should involve more ecologically valid combinations of tailored tasks.

## Data Availability

No datasets were generated or analysed during the current study.
